# Development of a biomarker mortality risk model in acute respiratory distress syndrome

**DOI:** 10.1186/s13054-019-2697-x

**Published:** 2019-12-16

**Authors:** Christian Bime, Nancy Casanova, Radu C. Oita, Juliet Ndukum, Heather Lynn, Sara M. Camp, Yves Lussier, Ivo Abraham, Darrick Carter, Edmund J. Miller, Armand Mekontso-Dessap, Charles A. Downs, Joe G. N. Garcia

**Affiliations:** 1 College of Medicine, University of Arizona Health Sciences, Tucson, AZ USA; 2 College of Pharmacy, University of Arizona Health Sciences, Tucson, USA; 3 PAI Life Sciences, Seattle, WA USA; 4 RDS2 Solutions, Stony Brook, NY USA; 5 Medical Intensive Care Unit, Henri Mondor Hospital, AP-HP, 94010 Créteil, France; 6 College of Nursing and Health Sciences, University of Florida, Gainesville, USA

**Keywords:** ARDS, Predictive analytics, Biomarkers, Mortality

## Abstract

**Background:**

There is a compelling unmet medical need for biomarker-based models to risk-stratify patients with acute respiratory distress syndrome. Effective stratification would optimize participant selection for clinical trial enrollment by focusing on those most likely to benefit from new interventions. Our objective was to develop a prognostic, biomarker-based model for predicting mortality in adult patients with acute respiratory distress syndrome.

**Methods:**

This is a secondary analysis using a cohort of 252 mechanically ventilated subjects with the diagnosis of acute respiratory distress syndrome. Survival to day 7 with both day 0 (first day of presentation) and day 7 sample availability was required. Blood was collected for biomarker measurements at first presentation to the intensive care unit and on the seventh day. Biomarkers included cytokine-chemokines, dual-functioning cytozymes, and vascular injury markers. Logistic regression, latent class analysis, and classification and regression tree analysis were used to identify the plasma biomarkers most predictive of 28-day ARDS mortality.

**Results:**

From eight biologically relevant biomarker candidates, six demonstrated an enhanced capacity to predict mortality at day 0. Latent-class analysis identified two biomarker-based phenotypes. Phenotype A exhibited significantly higher plasma levels of angiopoietin-2, macrophage migration inhibitory factor, interleukin-8, interleukin-1 receptor antagonist, interleukin-6, and extracellular nicotinamide phosphoribosyltransferase (eNAMPT) compared to phenotype B. Mortality at 28 days was significantly higher for phenotype A compared to phenotype B (32% vs 19%, *p* = 0.04).

**Conclusions:**

An adult biomarker-based risk model reliably identifies ARDS subjects at risk of death within 28 days of hospitalization.

## Introduction

Acute respiratory distress syndrome (ARDS) is a devastatingly-intense inflammatory lung disorder characterized by severe respiratory failure requiring mechanical ventilation with a high mortality rate of 30–40% [1]. ARDS exhibits clinical and biological heterogeneity with respiratory and multi-organ system failure in response to diverse inciting stimuli. For example, in the USA, sepsis and pneumonia are the major inducers of ARDS, whereas in India, ARDS occurs in response to various tropical infections (malaria, miliary tuberculosis, dengue infections, etc.) [2]. The clinical and radiographic diagnostic criteria for ARDS are relatively imprecise [3]. Diagnostic uncertainty in ARDS further exacerbates disease heterogeneity and is a potential source of bias in conducting clinical trials. Consequently, the development of novel therapies in ARDS has been extremely challenging and contributes to the abysmal track record of Phase II/III ARDS clinical trials [4]. Scoring systems in critically ill patients such as the APACHE II score [5] or the lung injury severity score [6] successfully link to patient outcomes but fail to provide consistent and accurate predictive estimates of the risk of death in patient populations with a specific disease process. General severity scores lack specificity by failing to distinguish sepsis, ARDS, or acute kidney injury and are not significantly different between ARDS survivors and non-survivors [7]. Furthermore, general severity scores do not have pathophysiologic input and, therefore, are unlikely to guide personalized therapies. Attempts to characterize predictors of death in ARDS by developing a prognostic index [8] remain controversial and have not yet been replicated or validated. Thus, there is a compelling unmet medical need to identify clinical and/or biochemical disease-specific parameters that risk-stratify patients for both accurate prognostication and clinical trial purposes. Stratification of ARDS patients with reliable biomarkers predictive of mortality would optimize participant selection for clinical trial enrollment by focusing on those most likely to benefit from new clinical interventions [9, 10].

We and others have used “omic” approaches and translational systems biology methods to identify a panel of plasma protein biomarkers with diverse biological mechanisms that suggest a possible association with ARDS mortality [11–22]. The objective of the current study was to utilize plasma protein biomarkers to derive a prognostic model for predicting ARDS patients least likely to survive to 28 days. We used latent class analysis and predictive analytics on eight plasma biomarkers collected from a well-phenotyped combined ARDS cohort. Biomarkers included cytokine-chemokines (interleukin-6 [IL-6], interleukin-8 [IL-8], interleukin-1 beta [IL-1B], and interleukin-1 receptor antagonist [IL-1RA]), dual-functioning cytozymes, i.e., cytokine/intracellular enzymes (macrophage migration inhibitory factor [MIF], nicotinamide phosphoribosyltransferase [eNAMPT]) and vascular injury markers (sphingosine 1-phosphate receptor 3 [S1PR3], angiopoietin-2 [Ang-2]). These analyses utilizing complementary analytical approaches, latent class analysis, classification and regression tree (CART) analysis, and rank aggregation were consistent and allowed the identification of six biomarkers that were most predictive of ARDS mortality.

## Materials and methods

### Source of data

Clinical data and plasma samples from 252 well-phenotyped ARDS patients including 203 patients enrolled in the Fluid and Catheter Treatment Trial (FACTT) study [23], and 49 ARDS patients enrolled at the University of Arizona and University of Illinois (IRB#1312168664R001 and #20120192 respectively) were studied.

### Participants

All patients with ARDS who met the diagnostic criteria per the American-European Consensus Conference (AECC) [24] or the Berlin definition [3] were included.

### Outcome

The primary outcome was mortality by day 28.

### Predictors

Initial (D0) plasma samples were taken within 48 h of meeting ARDS criteria, and for those who survived, a second sample was collected on the seventh day (D7) for biomarker measurements. The plasma levels of the following biomarkers were measured: IL-6, IL-8, IL-1RA, IL-1B, MIF, eNAMPT, S1PR3, and Ang-2.

### Blood collection and plasma biomarker measurements

Blood was collected within 48 h of ARDS onset (defined as time of meeting all Berlin Criteria) in EDTA-treated tubes, centrifuged within 1 h from sample collection (2000×*g* for 20 min, RCF) and stored at − 80 °C (− 70 °C for FACTT samples). Plasma concentrations of four biomarkers (IL-6, IL-8, IL-1B, IL-1RA) were measured in duplicate using a custom Bio-Plex Pro Human Cytokine 5-plex immunoassay (Bio-Rad, Hercules, CA) and Bio-Plex MAGPIX instrument following the manufacturer’s guidelines. Enzyme-linked immunosorbent assay (ELISA) techniques were utilized to quantify plasma levels of eNAMPT (an internally developed ELISA [25]), MIF (R&D System®, Minneapolis, MN), S1P3 [26], and Ang-2 (R&D System®, Minneapolis, MN) using commercially available ELISAs according to the manufacturer’s instructions.

### Statistical analysis

#### Exploratory analysis

Exploratory data analysis was performed for each biomarker (baseline and day 7). Median values and first and third quartiles were calculated for the continuous variables. Percentage values were computed and reported for categorical variables. The signed rank test was performed to assess the differences between initial biomarker measurements compared to those taken 7 days later.

#### Logistic regression

To identity biomarkers that potentially significantly contribute to mortality, binary logistic regression and a multiple forward stepwise logistic regression was constructed with APACHE, IL-6, IL-8, IL-1RA, IL-1B, MIF, eNAMPT, S1PR3, and Ang-2 as covariates. In addition, a binary logistic regression was performed, where mortality was fit to the difference in measurements (D0 and D7) for each biomarker to determine whether the mortality status of patients is affected by a change in individual biomarker measurements.

#### Latent class analysis

Eight plasma biomarkers (D0) were included as inputs in the latent class analysis (LCA) model. A series of latent class models were fitted with different class sizes, specifically, class sizes of two, three, four, and five. The criteria for selecting the optimal number of classes were based on Bayesian Information Criteria (BIC), the Vuong-Lo-Mendell-Rubin (VLMR) likelihood ratio test, and the size of the smallest class. Latent class model estimation was based on full-information maximum likelihood methods as implemented in Mplus [9]. To understand how the biomarkers distinguished each class, study participants were assigned to their most likely class and the mean values of the biomarkers compared by class assignment. Once the number of classes was determined, we tested the association between class assignment and 28-day mortality.

#### Classification and regression trees (CART)

CART analysis has been used in many areas for decision-making purposes to develop models that can classify and predict subjects into various risk categories [27]. We used CART analysis to determine the optimal combination of biomarkers and cut-offs predictive of mortality. The optimal classification tree was derived with a random 80% sampling of ARDS patients with D0 biomarker measurements, while imposing a condition to the CART algorithm of a minimum of 15 measurements needed to split a node. Terminal nodes that did not improve the classification based on class probability method were pruned. Weighting of cases and costs for misclassification were not used. The remaining 20% of patients with D0 biomarker measurements were used for testing. As the test data (50 patients) was underpowered to generate a meaningful CART algorithm, we used resampling techniques to create a new data consisting of 200 patients. This was performed by randomly selecting individuals with replacement. CART analysis was performed on the resampled data and test diagnostics calculated. Diagnostic statistics, presented as sensitivity, specificity, positive predictive value, and negative predictive value of the risk classification model, were computed separately for the derivation and test cohorts.

#### Ranking of biomarkers by importance after CART analysis

We tested ten iterations of the data in order to rank the biomarkers by importance to the classification trees: (1) all D0 and D7 biomarker measurements combined, (2) D0 biomarker measurements only, (3) first half of D0 biomarker measurements, (4) second half of D0 biomarker measurements, (5) first half of D7 biomarker measurements, (6) second half of D7 measurements, (7) D7 measurements only, (8) 80% random sample of D0 cohort. (Additional file 2: Table S1). For each iteration, the following conditions were imposed: (i) 15 or greater observations required for a nodal split and (ii) splits must decrease the overall lack of fit by a factor of 1e−5 (or 0.001%). The analyzed results from each of the 20 created CART outputs includes a list of biomarkers arranged by the importance to the classification tree along with an associated numeric score where an elevated score indicates increasing biomarker importance in predicting mortality. Finally, a rank aggregation algorithm [28] was used to generate final ranking of biomarkers by importance for each ordered list. All analyses were performed in R (R Core Team, 2018) and Mplus v8.3 [29].

## Results

### Characteristics of the cohort

The demographic and clinical characteristics of the ARDS subjects are presented in Table 1. Mortality among ARDS patients was 22% with non-survivors significantly older than survivors. There were no other significant differences in the clinical characteristics between ARDS survivors and non-survivors (Table 1). The medians (IQR) for all eight biomarkers for ARDS patients at day 0 and day 7 are presented in Table 2. Of the eight biomarkers, only eNAMPT was significantly higher on day 7 compared to day 0 (35% higher) (Table 2). Values of IL-6, IL-1RA, IL-8, Ang-2, and S1PR3 were significantly higher on day 0 compared to day 7. There was no significant difference between levels of MIF and IL-1B on days 0 and 7.

**Table 1:** Demographics and clinical characteristics of the ARDS cohort

Variable	ARDS cohort		*P* value
	Alive = 197	Dead = 55	
Sex, female, *N* (%)	100 (51%)	22 (40%)	0.16
Age, median (Q1, Q3)	48 (39, 56)	58 (49, 70)	< 0.0001
APACHE II Score, median (Q1, Q3)	76 (56, 101)	91 (28, 124)	0.10
Race/ethnicity, *N* (%)			
Black or African American	30 (15%)	16 (29%)	0.05
White	163 (83%)	39 (71%)	
Other*	4 (2.0%)	0 (0%)	
ARDS etiology, *N* (%)			
Sepsis	89 (45%)	31 (56%)	0.14
Trauma	17 (8.6%)	5 (9%)	1.0
Pneumonia	127 (64%)	37 (67%)	0.7
Source of cohort data			
FACTT cohort	162	41	
University of Illinois cohort	8	9	
University of Arizona cohort	27	5	

*Other indicates: any race/ethnicity other than Black or White

*FACTT* Fluids and Catheters Treatment Trial

**Table 2:** Biomarker plasma levels at day 0 and at day 7 in ARDS cohort

Biomarker	D0 median (Q1, Q3) (*N*)	D7 median (Q1, Q3) (*N*)	*P* value^1^
IL-6 (pg/ml)	27 (7, 92) 145	12 (4, 54) 115	< 0.01
IL-8 (pg/ml)	150 (67, 359) 201	108 (44, 298) 200	0.001
IL-1RA (pg/ml)	3072(1241, 6545) 229	2755 (1067, 5695) 225	0.04
MIF (ng/ml)	45(29, 78) 242	49 (31, 84) 247	0.07
NAMPT (ng/ml)	60 (34, 74) 248	81 (55, 113) 248	< 0.001
S1PR3 (ng/ml)	577(227, 1458) 231	399 (97, 1066) 233	< 0.001
Ang-2 (ng/ml)	12 (7, 23) 247	7 (4, 12) 247	< 0.001
IL-1B (pg/ml)	44(24, 68) 228	39 (20, 61) 233	< 0.01

^1^*P* value from Wilcoxon signed rank test comparing the measurements for D0 and D7 for all eight biomarkers. *IL-6* interleukin-6, *IL-8* interleukin-8, *IL-1RA* interleukin-1 receptor antagonist, *MIF* Macrophage migration inhibitory factor, *NAMPT* nicotinamide phosphoribosyltransferase, *S1PR3* sphingosine 1-phosphate receptor 3, *Ang-2* angiopoietin-2, *IL-1B* interleukin 1 beta

### Results of logistic regression

Logistic regression analysis assessing the ability of each biomarker measured at D0 to predict mortality failed to show any biomarker to be statistically significant after adjustment for covariates **(**Additional file 2: Table S2**).** For measurements taken at D7, only Ang-2 levels were significantly associated with an increase in mortality **(**Additional file 2: Table S3**).** There was no significant association between the change in biomarker measurements taken at the two time points and mortality **(**Additional file 2: Table S4**).**

### Latent-class modeling and characteristics of each phenotype

**Fig. 1:**
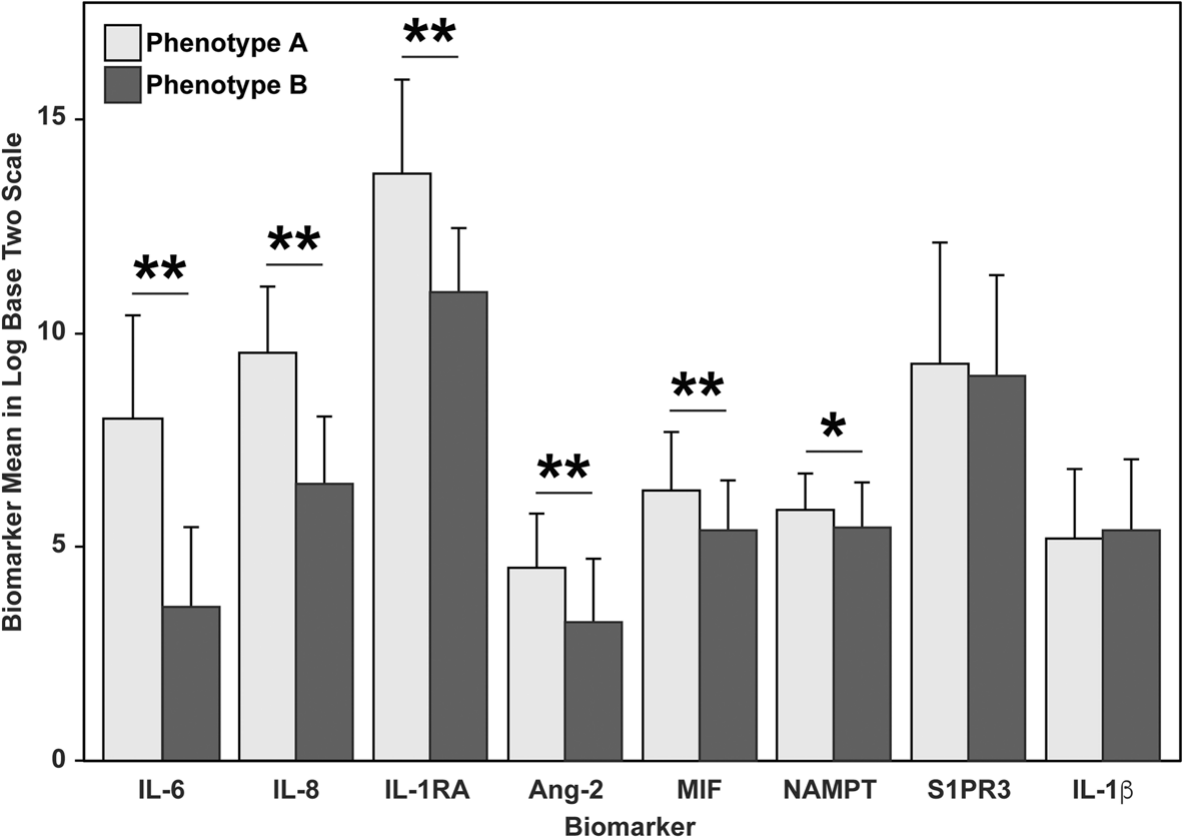
Differences in biomarker levels by phenotype. Graph showing estimated means for eight biomarkers. Phenotype A is characterized by higher levels of Ang-2, MIF, IL-8, IL-1RA, IL-6, and NAMPT compared to phenotype B

A summary of the model fits for two through five classes is presented in Additional file 2: Table S5 with a two class model for the eight biomarkers providing the optimal fit. The average latent class probabilities were 0.77 for phenotype B and 0.23 for phenotype A **(**Additional file 1: Figure S1**)**. Compared to phenotype B, phenotype A exhibited considerably higher plasma levels of Ang-2, IL-6, eNAMPT, MIF, IL-8, and IL-1RA **(**Fig. 1**).** There was no significant phenotype difference between levels of IL-1B and S1PR3. In order to determine whether the two phenotypes exhibit differing natural histories, we tested the association between each phenotype and 28-day mortality. Phenotype A subjects exhibited significantly higher mortality compared to phenotype B subjects (32% vs 19%, *p* = 0.04).

### Derivation and testing of classification tree

**Fig. 2:**
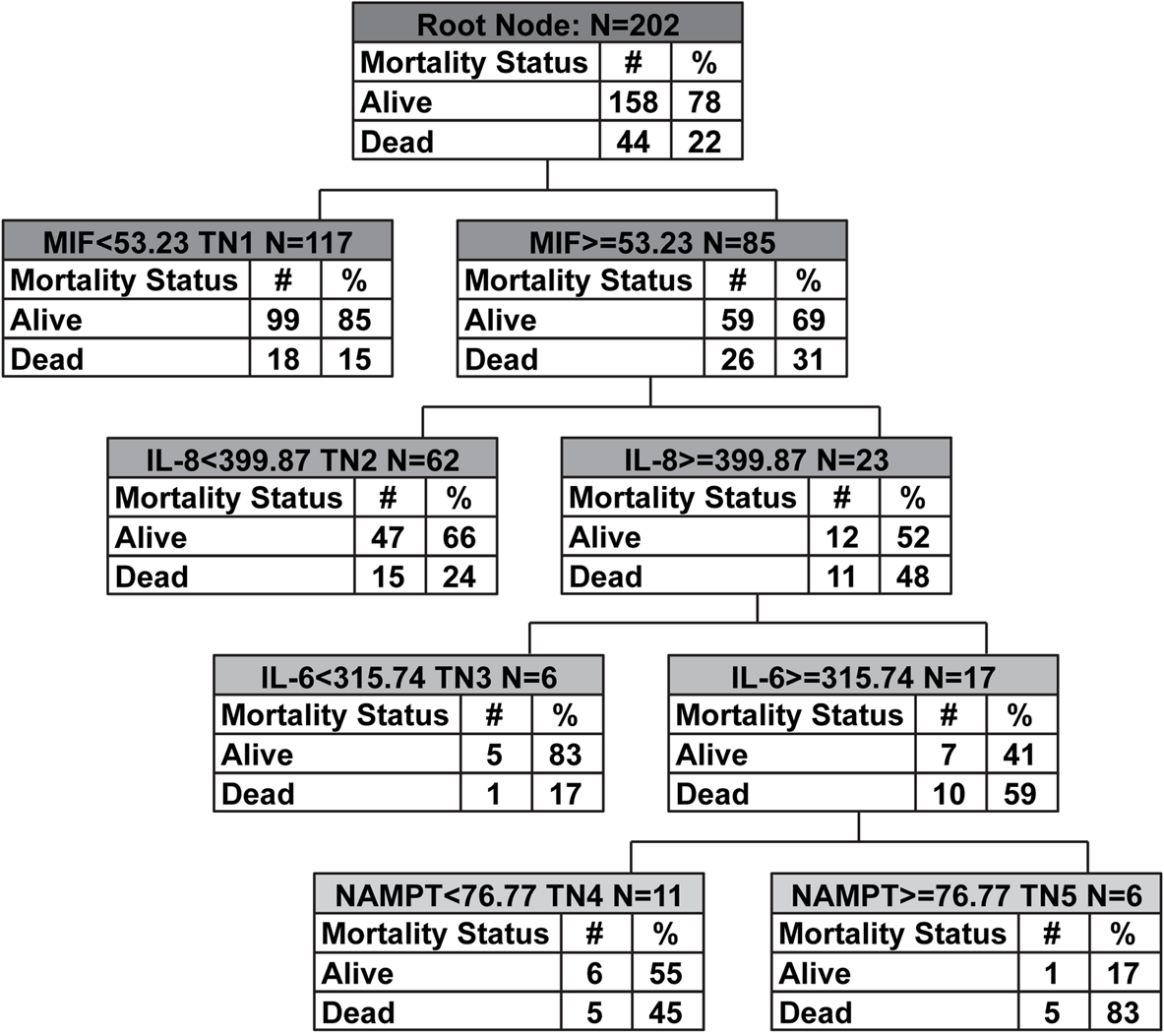
Biomarker-based ARDS Mortality Risk Stratification Model decision tree. The derived biomarker-based ARDS Mortality Risk Stratification Model decision tree from the random 80% derivation cohort (*n* = 202, 44 non-survivors). The tree contains the mortality probability, macrophage migration inhibitory factor (MIF), interleukin 8 (IL-8), interleukin 6 (IL-6), and extracellular nicotinamide phosphoribosyltransferase (NAMPT). Biomarker concentrations are expressed in ng/ml. Terminal nodes (TN) 1 and TN3 were low risk (15% and 17% risk of death), TN2 and TN4 were intermediate risk (24% and 45% risk of death, respectively), and TN5 high risk (85% risk)

Maximum accuracy for predicting ARDS mortality was achieved with four of the eight candidate biomarkers: MIF, IL-8, IL-6, and eNAMPT **(**Fig. 2**)**. No single demographic or clinical variable improved ARDS mortality prediction accuracy. Two low risk nodes (TN1 and TN3, mortality 15% and 17% respectively), two medium risk nodes (TN2 and TN4, mortality 24% and 45%, respectively), and one high risk node (TN5 with mortality of 83%) were identified. The test characteristics of the biomarker-based ARDS Mortality Risk Stratification Model decision trees are shown in Table 3.

**Table 3:** Test characteristics of the biomarker-based ARDS Mortality Risk Stratification Model decision trees

Variables	Derivation, 80% day 0, (*n* = 202, 44 non-survivors)		Test, 20% day 0, resampled to *n* = 200	
	Values	95% CI	Values	95% CI
True negatives	5	–	28	–
False positives	19	–	7	–
True positives	104	–	131	–
False negatives	1	–	8	–
Sensitivity	0.99	(0.95, 0.99)	0.94	(0.88, 0.97)
Specificity	0.21	(0.07, 0.42)	0.80	(0.63, 0.97)
Positive predictive value	0.85	(0.81, 0.87)	0.95	(0.91, 0.97)
Negative predictive value	0.83	(0.39, 0.98)	0.78	(0.64, 0.87)

### Ranking of biomarkers by importance

Output from CART analysis allowed for a ranking of biomarkers by order of importance in terms of predicting mortality resulting in identification of the top six biomarkers: Ang-2, MIF, IL-8, IL-1RA, IL-6, and eNAMPT (Additional file 2: Table S6).

## Discussion

We initially included eight ARDS-relevant plasma biomarkers derived from specific pathobiologic groups including: (i) inflammatory cytokine-chemokines (IL-6, IL-8, IL-1B, and IL-1RA), (ii) dual-functioning cytozymes, i.e., an intracellular enzyme that also functions as a cytokine when secreted (macrophage migration inhibitory factor, eNAMPT), and iii) vascular injury markers (S1PR3, Ang-2). Our findings indicate that a panel of six biomarkers potentially predict ARDS survival. In the latent class modeling we performed, a biomarker-based phenotype with higher plasma levels of Ang-2, MIF, IL-8, IL-1RA, IL-6, and eNAMPT was associated with significantly higher mortality **(**Fig. 1**)**. The complementary CART analysis revealed that the maximum accuracy for predicting high mortality was achieved by four out of these six biomarkers (MIF, IL-8, IL-6, and eNAMPT) (Fig. 2). Finally, rank aggregation identified Ang-2, MIF, IL-8, IL-1RA, IL-6, and eNAMPT as the most important in contributing to mortality (Additional file 2: Table S6).

The pathogenesis of ARDS includes a combination of endothelial injury, epithelial injury, an intense inflammatory cascade, dysregulated coagulation, fibrosis, and apoptosis in response to diverse stimuli. Studies exploring the byproducts of acute dysregulation of various cellular pathways have generated more than 45 potential biomarkers [30, 31]. However, no single biomarker or clinical variable has demonstrated adequate prognostic or predictive ability to identify sub-phenotypes of ARDS [9, 10, 32, 33]. The ability to stratify ARDS patients by pathobiology and likelihood of treatment response would greatly enrich future clinical trials and enhance the ability to detect a treatment effect [34]. Sub-phenotyping/endotyping has been successfully accomplished in airways diseases such as asthma and COPD with important therapeutic implications [35, 36] and may exist within severe sepsis [4, 37, 38]. However, there is a paucity of data elucidating ARDS sub-phenotypes/endotypes. Recent studies [9, 39], utilizing two ARDSnet cohorts, identified two ARDS sub-phenotypes that markedly differed in natural history, clinical and biological characteristics, biomarker profiles, response to positive end-expiratory pressure (PEEP), and ventilator- and organ failure free days and in mortality. The hyperinflammatory ARDS sub-phenotype is characterized by a higher prevalence of sepsis and severe shock, high plasma levels of inflammatory biomarkers (IL-6, IL-8, etc.), greater vasopressor use, and metabolic acidosis. In contrast, the low inflammatory ARDS sub-phenotype exhibited less severe inflammation and shock. Surprisingly, the level of ARDS severity (PaO2/FiO2 ratio), renal or hepatic injury severity, or leukocytosis level failed to distinguish these two phenotypes [9]. The hyperinflammatory phenotype was associated with higher mortality, fewer ventilator-free and organ failure-free days, and altered responses to ventilator strategies when compared to the low inflammatory phenotype [40]. Importantly, no single clinical or biological variable was sufficient to identify the sub-phenotype including the severity of ARDS APACHE scores (PaO2/FiO2 ratio), severity of renal or hepatic failure, or leukocytosis, suggesting that phenotype membership was not merely a reflection of severity of illness as measured by traditional indices [40].

Our findings are strengthened by the use of three complementary approaches, including predictive analytics, to identify a common biomarker combination that is important in predicting ARDS survival. We also used a well-phenotyped cohort of ARDS patients, the majority of whom were enrolled as part of a large multicenter trial. Therefore, the participants represent a demographically diverse cohort of patients with ARDS strengthening the generalizability of our findings.

Our study has several limitations including the relative low observed mortality of 22% in the examined cohort compared to the expected ARDS mortality of 30–40%. This is likely due to the selection criteria we utilized where only patients surviving to day 7 were included in order to analyze both days 0 and 7 samples, i.e., patients who died before day 7 were not included. A second limitation of our work is that we had limited statistical power to detect significant differences in the predictive modeling algorithms. Finally, we recognize the lack of an external validation of our ARDS biomarker-based risk model in an independent cohort. Clearly, despite our utilization of a random sample of 80% of ARDS patients at D0 for the derivation and resampling technique on the remaining 20% of patients to demonstrate good test characteristics **(**Table 3**)**, we plan to next evaluate our mortality panel an independent cohort to determine the reproducibility of these six biomarkers in predicting ARDS survival.

## Conclusion

We have used complementary analytic approaches to identify six biomarkers (Ang-2, MIF, IL-8, IL-1RA, IL-6, eNAMPT) that show promise in predicting survival in ARDS. These biomarkers provide an important proof-of-concept that a combination of ARDS biomarkers can improve sub-phenotyping and enhance predictive and prognostic ability at the time of diagnosis. This information could be useful for guiding enrollment in clinical trials and represents an important step towards better patient stratification at the time of presentation to enhance the likelihood of a positive clinical trial in this vexing disorder.

## Trial status

Not applicable.

## Supplementary information

**Additional file 1: Figure S1.** Probability of Latent Class Assignment. Barplot of probability of class assignment using eight biomarkers.

**Additional file 2: Table S1.** Ranking algorithm. Eight CART iterations, algorithm conditions imposed, and number of biomarkers used. **Table S2.** Logistic regression analysis of mortality prediction capacity of the eight ARDS biomarkers-Day Zero. **Table S3.** Logistic regression analysis of mortality prediction capacity of the eight ARDS biomarkers-Day Seven. **Table S4.** Logistic regression analysis to assess the mortality prediction for change in measurements for the eight biomarkers. **Table S5.** Number of individuals per latent class. **Table S6.** Ranking of ARDS biomarker importance in predicting mortality.

## Data Availability

Yes. Primary source data and methods are available.
