# Supplementary figures and images for: Development of a biomarker mortality risk model in acute respiratory distress syndrome
Source: Crit Care. 2019 Dec 16;23:410. doi: 10.1186/s13054-019-2697-x (PMC6916252; doi:10.1186/s13054-019-2697-x)

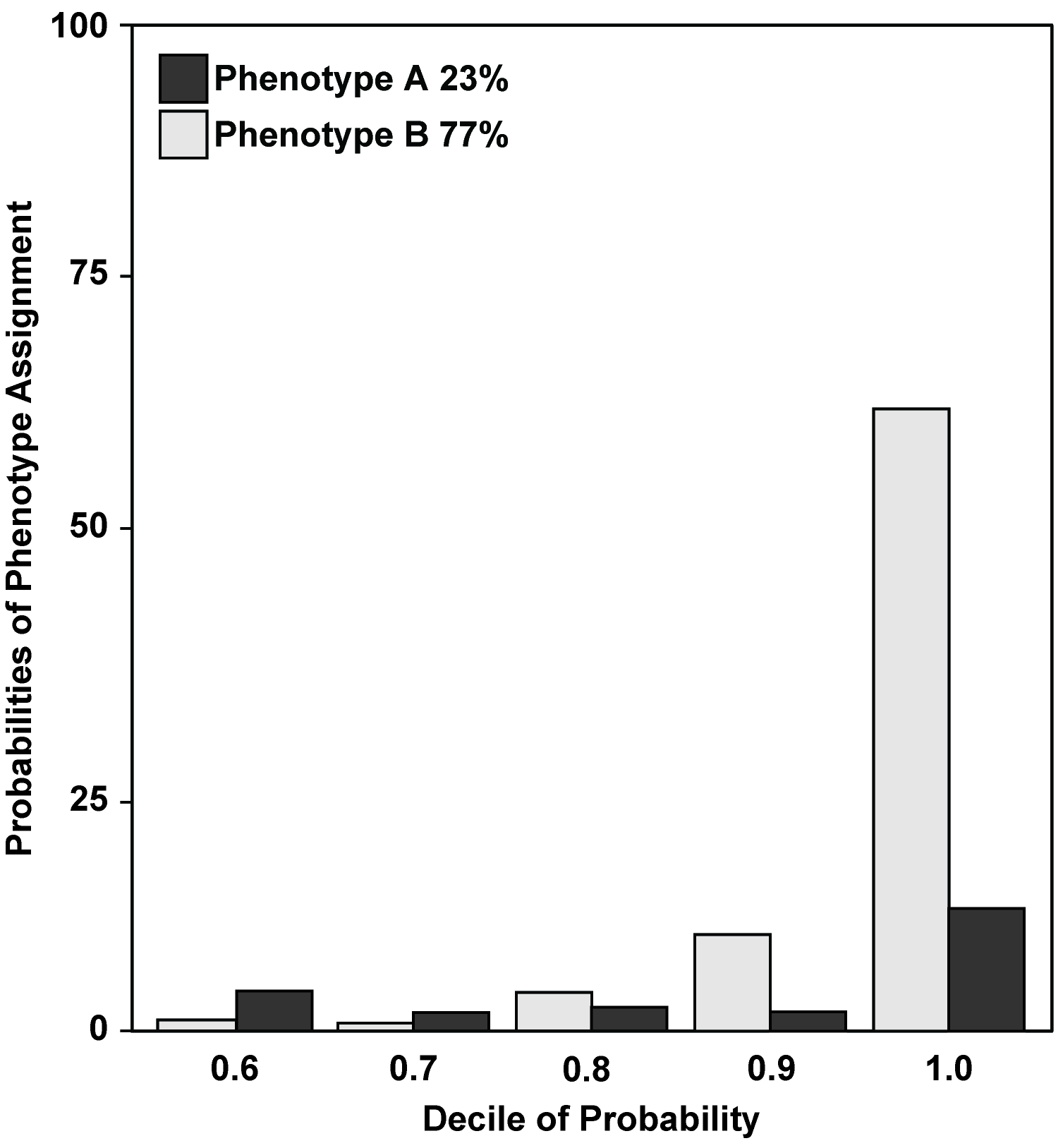

Supplement: Supplementary file 1 — Additional file 1: Figure S1. Probability of Latent Class Assignment. Barplot of probability of class assignment using eight biomarkers. [file 13054_2019_2697_MOESM1_ESM.tif]
