# Supplementary material for: Development of a biomarker mortality risk model in acute respiratory distress syndrome
Source: Crit Care. 2019 Dec 16;23:410. doi: 10.1186/s13054-019-2697-x (PMC6916252; doi:10.1186/s13054-019-2697-x)
Supplement: Supplementary file 2 — Additional file 2: Table S1. Ranking algorithm. Eight CART iterations, algorithm conditions imposed, and number of biomarkers used. Table S2. Logistic regression analysis of mortality prediction capacity of the eight ARDS biomarkers-Day Zero. Table S3. Logistic regression analysis of mortality prediction capacity of the eight ARDS biomarkers-Day Seven. Table S4. Logistic regression analysis to assess the mortality prediction for change in measurements for the eight biomarkers. Table S5. Number of individuals per latent class. Table S6. Ranking of ARDS biomarker importance in predicting mortality. [file 13054_2019_2697_MOESM2_ESM.docx]

**Additional file 2**

**TABLE S1. Ranking algorithm. Eight CART iterations, algorithm conditions imposed, and number of biomarkers used.**

| **CART iteration and data used** | **Number of biomarkers used by CART algorithm** | **Conditions imposed** |
| --- | --- | --- |
| All D0 & D7 minus controls | 10 | One |
| All D0 & D7 minus controls | 11 | One & Two |
| D0 only | 09 | One |
| D0 only | 11 | One & Two |
| First half D0 only | 06 | One |
| First half D0 only | 09 | One & Two |
| Second half D0 only | 05 | One |
| Second half D0 only | 09 | One & Two |
| First half D7 only | 05 | One |
| First half D7 only | 07 | One & two |
| Second half D7 only | 06 | One |
| Second half D7 only | 11 | One & two |
| D7 only | 09 | One |
| D7 only | 10 | One & Two |
| 80% random sample D0 | 06 | One |
| 80% random sample D0 | 10 | One & Two |

**TABLE S1.** Details of ranking methodology for biomarker importance. Column 1 has the eight iterations of the data used to rank the biomarkers by importance to the classification trees. There are 20 total rows for each iteration reflecting that either one or two conditions were imposed on the CART algorithm. The first and default condition was that at least 15 observations are required in order for a split to occur at a node. The second condition is that a split must decrease the overall lack of fit by a factor of 1e-5 (or 0.001%) – see column 3. The middle column indicates the number of biomarkers ultimately used for each iteration.

**TABLE S2. Logistic regression analysis of mortality prediction capacity of the eight ARDS biomarkers-Day Zero.**

| **Biomarker** | **Odds Ratio** | **P-value** | **Adjusted P-value** |
| --- | --- | --- | --- |
| NAMPT | 1.00 | 0.733 | 0.932 |
| IL-1RA | 1.00 | 0.820 | 0.932 |
| IL-6 | 1.00 | 0.351 | 0.656 |
| IL-8 | 1.00 | 0.286 | 0.656 |
| Ang-2 | 1.01 | 0.164 | 0.656 |
| IL-1B | 1.00 | 0.410 | 0.656 |
| MIF | 1.00 | 0.932 | 0.932 |
| SIPR3 | 1.00 | 0.237 | 0.656 |

**TABLE S3. Logistic regression analysis of mortality prediction capacity of the eight ARDS biomarkers-Day Seven.**

| **Biomarker** | **Odds Ratio** | **P-value** | **Adjusted P-value** |
| --- | --- | --- | --- |
| eNAMPT | 1.00 | 0.827 | 0.827 |
| ILRA | 1.00 | 0.149 | 0.298 |
| IL6 | 1.00 | 0.580 | 0.701 |
| IL8 | 1.00 | 0.092 | 0.298 |
| ANG-2 | 1.08 | 0.000 | 0.000 |
| IL-1B | 1.00 | 0.468 | 0.701 |
| MIF | 1.00 | 0.614 | 0.701 |
| SIPR3 | 1.00 | 0.143 | 0.298 |

**TABLE S4. Logistic regression analysis to assess the mortality prediction for change in measurements for the eight biomarkers.**

| **Biomarker** | **Odds Ratio** | **P-value** | **Adjusted P-value** |
| --- | --- | --- | --- |
| eNAMPT | 1.00 | 0.923 | 0.923 |
| ILRA | 1.00 | 0.380 | 0.923 |
| IL6 | 1.00 | 0.722 | 0.923 |
| IL8 | 1.00 | 0.670 | 0.923 |
| ANG-2 | 1.00 | 0.031 | 0.246 |
| IL-1B | 1.00 | 0.587 | 0.923 |
| MIF | 1.00 | 0.902 | 0.923 |
| S1PR3 | 1.00 | 0.087 | 0.346 |

**TABLE S5. Number of individuals per latent class**

| **Number of Classes** | **Bayesian Information Criteria** | **Entropy** | **Number of Patients Assigned to Each Class** | | | | | **P-value** |
| --- | --- | --- | --- | --- | --- | --- | --- | --- |
|  |  |  | N_1_ | N_2_ | N_3_ | N_4_ | N_5_ |  |
| 2 | 6916.715 | 0.734 | 195 | 57 |  |  |  | 0.2877 |
| 3 | 6872.799 | 0.835 | 20 | 191 | 41 |  |  | 0.2829 |
| 4 | 6887.425 | 0.792 | 11 | 112 | 7 | 112 |  | 0.3302 |
| 5 | 6860.447 | 0.789 | 11 | 97 | 7 | 30 | 107 | 0.6340 |

**TABLE-S6. Ranking of ARDS biomarker importance in predicting mortality.**

| **Order** | **D0, D7 data** | **Composite ranking^§^** |
| --- | --- | --- |
| 1 | MIF | Ang-2 |
| 2 | IL-6 | MIF |
| 3 | Ang-2 | IL-8 |
| 4 | IL-8 | IL-6 |
| 5 | NAMPT | IL-1RA |
| 6 | IL-1B | NAMPT |
| 7 | IL-1RA | S1PR3 |
| 8 | S1PR3 | IL-1B |

^§^All CART outputs

Ranking of biomarkers by importance in terms of contribution to the classification tree and mortality. The first column is the rank order by decreasing importance based on various data iterations used in CART input. In column 2, ranking is based on CART analysis of D0, D7 data. Column 3 is a composite ranking from all CART iterations. The top biomarkers based on rank aggregation are Ang-2, MIF, IL-8, IL-1RA, IL-6, and NAMPT.
